# Differential sperm‐egg interactions in experimental pairings between two subspecies and their hybrids in a passerine bird

**DOI:** 10.1002/ece3.4624

**Published:** 2018-10-31

**Authors:** Laura L. Hurley, Melissah Rowe, Simon C. Griffith

**Affiliations:** ^1^ Department of Biological Sciences Macquarie University Sydney New South Wales Australia; ^2^ Natural History Museum, University of Oslo Oslo Norway; ^3^ Centre for Ecological and Evolutionary Synthesis, Department of Biosciences University of Oslo Oslo Norway

**Keywords:** finch, hybridization, perivitelline layer, postmating prezygotic reproductive barriers, speciation

## Abstract

Speciation research has largely overlooked reproductive barriers acting between copulation and the formation of the zygote (i.e., postmating, prezygotic [PMPZ] barriers), especially in internally fertilizing vertebrates. Nonetheless, it is becoming clear that PMPZ reproductive barriers can play a role in the formation and maintenance of species boundaries. We investigated sperm‐egg interactions in the recently diverged subspecies pairs of the long‐tailed finch, *Poephila acuticauda acuticauda* and *P. a. hecki*, to explore potential PMPZ barriers. Specifically, we compared the number of sperm reaching the perivitelline layer (PVL) of the ova, and hence the site of fertilization, in both intra‐ and inter‐subspecies pairings and pairings of F1 hybrid adults with one parental subspecies. Although we found no difference in PVL sperm number among intra‐ and inter‐subspecific pairs, a significantly lower number of sperm reached the site of fertilization in a backcross pairing. As low numbers of PVL sperm appear to be associated with low fertilization success in birds, our findings offer insight into the potential role of postcopulatory processes in limiting gene flow between the subspecies and may help explain the relatively narrow hybrid zone that exists in the wild in this species. Though further work is needed to gain a comprehensive understanding of the morphological, physiological, and molecular mechanisms underlying our results, our study supports the role of PMPZ reproductive barriers in avian speciation, even in recently diverged taxa, that may not yet be fully genetically incompatible.

## 1 INTRODUCTION

Recent research supports the idea that sexual selection promotes species divergence (reviewed in Kraaijeveld, Kraaijeveld‐Smit, & Maan, 2011, Ritchie, 2007). Generally speaking, however, this work has overwhelmingly investigated precopulatory sexual selection, whereas the consequences of postcopulatory sexual selection to the speciation process have been largely overlooked (reviewed in Coyne & Orr, 2004, Howard, Palumbi, Birge, & Manier, 2009, Ritchie, 2007). As such, the study of speciation has neglected reproductive barriers acting between copulation and the formation of the zygote (i.e., postmating, prezygotic barriers, PMPZ; Coyne & Orr, 2004; Howard et al., 2009). This is particularly true for internally fertilizing species and is likely attributable to the cryptic and complex nature of ejaculate‐female and sperm‐egg interactions in such taxa (Howard et al., 2009; Pitnick, Wolfner, & Suarez, 2009). Nonetheless, postcopulatory sexual selection is an important evolutionary force capable of driving rapid evolutionary change in female and male reproductive traits, including sperm (Pitnick, Wolfner, et al., 2009; Rowe, Albrecht, et al., 2015; Swanson & Vacquier, 2002), and there is growing recognition that PMPZ reproductive barriers have enormous potential to influence the formation and maintenance of species boundaries (McDonough, Whittington, Pitnick, & Dorus, 2016; Turissini, McGirr, Patel, David, & Matute, 2018).

Spermatozoa exhibit enormous morphological diversity across the animal kingdom (Pitnick, Hosken, & Birkhead, 2009). Though the role of sperm in reproductive isolation is not well understood, it has been suggested that divergence in sperm traits between genetically distinct allopatric populations can lead to compromised ejaculate‐female interactions upon secondary contact and, ultimately, PMPZ reproductive isolation (Howard et al., 2009). Such postcopulatory processes may include conspecific sperm precedence (i.e., the ability of conspecific sperm to fertilize eggs more efficiently than hetero‐specific sperm under competitive conditions) or cryptic female choice (i.e., where the female discriminates against hetero‐specific sperm; Ball & Parker, 2003; Griffith & Immler, 2009; Howard, 1999). Recent studies in *Drosophila* (Lüpold et al., 2012; Manier et al., 2013) and mice (Albrechtová et al., 2012; Dean & Nachman, 2009) build support for these ideas.

Evidence that ejaculate‐female/sperm‐egg interactions can impact hetero‐specific fertilization in avian systems comes primarily from experimental studies of Galliformes (turkey, chicken, etc) and Anseriformes (ducks, etc). For example, inseminations of hetero‐specific sperm resulted in reduced sperm storage, relative to conspecific inseminations, in chicken/turkey (Steele & Wishart, 1992) and common duck/Muscovy duck (Sellier et al., 2005) crosses. In the latter cross, hetero‐specific inseminations also resulted in an increase in the proportion of infertile eggs relative to conspecific inseminations (Sellier et al., 2005). Reports of reduced fertility in a number of studies offer some additional support for PMPZ barriers to hetero‐specific sperm (reviewed in Birkhead & Brillard, 2007), but the majority of these studies are unable to clearly distinguish between PMPZ and postzygotic (e.g., early embryo mortality) barriers and focus primarily on intergeneric crosses.

While studies of passerines with respect to PMPZ are relatively uncommon, in the domesticated Gouldian finch, *Erythrura gouldiae*, there is evidence for genetic incompatibility between the two common head color morphs in the laboratory (Pryke & Griffith, 2009), and experimental work found evidence of significant biases in fertilization favoring sperm from a female's own morph rather than the alternative morph (Pryke, Rollins, & Griffith, 2010). Although this work was unable to identify a mechanism, a recent study of sister species of naturally hybridising, but incompatible *Ficedula* flycatchers found that oviductal fluid collected from female pied flycatchers, *Ficedula hypoleuca*, negatively impacts hetero‐specific sperm performance (i.e., collared flycatcher, *F. albicollis*) under in vitro conditions (Cramer, Ålund, McFarlane, Johnsen, & Qvarnström, 2016).

A major challenge for the study of PMPZ barriers in internally fertilizing species is the difficulty in observing processes occurring within the female reproductive tract (Howard et al., 2009; Pitnick, Hosken, et al., 2009), which is especially true for vertebrates. In birds, however, the ubiquity of polyspermic eggs and the existence of a well‐established method to examine the number of sperm that reach and penetrate the perivitelline layer (PVL) of the ovum (e.g., Hurley, Fanson, & Griffith, 2017) provides a unique and non‐invasive approach to the study of PMPZ barriers. Importantly, in birds, fertilization rate increases as the number of sperm incorporated into the egg is higher (Birkhead & Fletcher, 1998; Mizushima, 2017), but too many sperm increase the likelihood of early embryonic failure (Christensen, Fairchild, & Ort, 2005; Forstmeier & Ellegren, 2010). As such, either low or high numbers of sperm present on the egg PVL may be linked to reduced reproductive success.

Here, we examined the outcome of both intra‐ and inter‐subspecific crosses, as well as backcrosses between hybrids and one of the parental forms, in the two subspecies of long‐tailed finch, *Poephila acuticauda*, with respect to the number of sperm trapped by the outer PVL of the ova. The long‐tailed finch is a small Estrildid (Passeriformes: Passeroidea) finch endemic to northern Australia. Two subspecies, differentiated by bill color, are recognized: the yellow‐billed *P. a. acuticauda* in the west, and the red‐billed *P. a. hecki* in the eastern part of the species’ range (Higgins, Peter, & Cowling, 2006). The subspecies are thought to have diverged in allopatry between 0.3 to 0.57 million years ago (Jennings & Edwards, 2005; Singhal et al., 2015), with secondary contact estimated to have occurred between 21–14 kya ago (Fitzsimmons et al., 2013; Reeves et al., 2013). The current distribution of the species includes a relatively narrow (~150 km in width) zone of overlap where the subspecies interbreed and produce orange‐billed hybrid offspring (Griffith & Hooper, 2017). However, geographic cline analysis using data on bill coloration suggests there is selection acting against hybrids resulting in limited gene flow between the subspecies and thus maintenance of the subspecies as distinct, independently evolving populations (Griffith & Hooper, 2017). Here, we are examining the hypothesis that gene flow between the two subspecies is limited by the reduced mating success of mixed pairs or the pairing between a hybrid and one of the parental forms (the latter being the most direct route through which admixture between the two subspecies will occur). Importantly, males from the two subspecies exhibit significant differences in sperm length (Rowe, Griffith, Hofgaard, & Lifjeld, 2015), a trait linked to differential fertilization success in passerine birds (Bennison, Hemmings, Slate, & Birkhead, 2015). We therefore tested for differences in the total number of sperm reaching the egg at the time of fertilization among different experimental crosses, including intra‐ and inter‐subspecific and backcross pairs, to examine the potential contribution of ejaculate‐female/sperm‐egg interactions in reproductive isolation between these subspecies.

## 2 MATERIALS AND METHODS

The long‐tailed finches used in this study were part of a large captive population maintained at Macquarie University since being brought into captivity from the wild in 2009 and 2010. The focal individuals were either wild‐caught birds or first generation (F1) captive‐bred birds and included males and females of *P. a. acuticauda* (yellow billed, hereafter yellow or Y), *P. a. hecki *(red billed, hereafter red or R), and F1 hybrids (*acu* x *hecki*; orange billed, hereafter orange or O). Previous work has shown that *P. a. acuticauda *and *P. a. hecki* show significant differences in total sperm length, but that wild‐caught and captive‐bred individuals do not differ in sperm morphology (Rowe, Griffith, et al., 2015), and it also the case that in the closely related zebra finch that has been more extensively sampled, there is no difference in sperm morphology between wild and captive birds (Immler, Griffith, Zann, & Birkhead, 2012). All birds were sexually mature adults, had prior breeding experience, and, to the best of our knowledge, were unrelated (with the exception of two female siblings that were used in the experiment).

We established 33 breeding pairs during February‐June and September‐October 2014, representing the following pair types using a classical forward‐genetic cross design (female color listed first): red‐red (RR, *n* = 5), yellow‐yellow (YY, *n* = 6), yellow‐red (YR, *n* = 5), red‐yellow (RY, *n* = 6), orange‐red (OR, *n* = 5), and red‐orange (RO, *n* = 6). For logistical reasons related to the availability of birds and aviaries, we focused all of our attention on the backcross between hybrids and just one of the parental forms (red) and did not attempt to explore the reciprocal pairing (i.e., orange backcrossed to a yellow parental). For similar reasons, we did not focus on pairings between two hybrids (i.e., OO) as such pairs are likely to be of less consequence in the consideration of gene flow from one subspecies to the other.

Each pair was housed in an outdoor aviary (approximately 4 × 1.8 × 2.2 m) provided with four nest boxes, nesting material, and ad libitum food (dry finch seed) and water. We also provided daily supplemental foods (green pea mixtures and Queensland fruit fly, *Bactrocera tryoni*, pupae to encourage breeding. All work was conducted according to relevant national and international guidelines and was approved by the Macquarie University Animal Ethics Committee (Animal Research Authority 2013/28).

Nest boxes were checked daily for evidence of nest building or egg laying, and pairs were allowed to produce two full clutches of eggs. For each clutch, eggs were collected on the day they were laid and replaced with a dummy egg to encourage clutch completion. Once the clutch was complete (i.e., minimum of three eggs with no new eggs laid for 2 days), dummy eggs were left in the nest for a further 5 days to ensure pair was committed to the clutch and to normalize spacing in relaying. After the 5 days (during which time parents started to incubate the dummy eggs), all dummy eggs were removed to promote the production of a second clutch. Upon collection, eggs were maintained at 4ºC until the clutch was complete. For eggs collected in February‐June, eggs were stored at 4ºC until processing, while eggs collected in September‐October were frozen and stored at −20°C until processing. Previous work has shown that this slight difference in methodology has no impact on the appearance or structure of the perivitelline layer or the sperm trapped on the PVL (Hurley et al., 2017).

Analysis of the number of sperm trapped on the outer perivitelline layer (PVL) followed standard methods (e.g., Hemmings & Birkhead, 2015, Hurley et al., 2017). Briefly, we dissected the egg with fine scissors with the shell and albumen discarded. The intact yolk was then cut approximately in half with the germinal disk (GD) centerd on one half, the entire PVL removed and cleaned in phosphate buffered saline at 25°C. Both halves of the PVL were placed on a microscope slide and incubated with 15 µl of Hoechst 33,342 fluorescent dye (Sigma, 5 µg/ml). We systematically scanned and counted all sperm on both halves of the PVL at 200X using a fluorescence microscope (Leica DM5000 B). A number of holes in the inner PVL (i.e., holes caused by sperm penetration of the PVL) were not counted because previous work has shown that the number of holes on the inner PVL is correlated with the number of sperm on the outer PVL (e.g., Birkhead, Sheldon, & Fletcher, 1994).

### 2.1 Data analysis

All statistical analysis was performed using R (version 3.3.3; R Core Team, 2017) *lme4* package (Bates, Machler, Bolker, & Walker, 2015) with p‐values calculated via *lmerTest* (Kuznetsova, Brockhoff, & Christensen, 2016). We investigated the impact of experimental pairings on the number of sperm reaching the egg PVL using negative binomial generalized linear mixed models (GLMMs) and running pairwise comparisons of all pair types. We first ran a model which included only intra‐ and inter‐subspecific pairs (i.e., RR, YY, RY, YR). A second model included a comparison of the two F1‐hybrid backcross pairings (OR, RO) and the parental intra‐subspecific pair (i.e., RR). In both models, we started with an interaction of the fixed effects, pair type and clutch, but they were not significant in either model (|z| > 0.01, *p* > 0.49) so were not included in final models. In both models, random effects included female identity and male identity to account for the use of some individuals in more than one pair (which was necessary due to limited bird numbers), and the random effect of pair identity had random intercepts as well as slopes by using an interaction with the order effect of clutch. To perform pairwise post hoc comparisons of all pair types, we iteratively changed the reference levels of the variable (i.e., pair type) to determine the estimated contrasts for all levels of the variable. We applied false discovery rate correction for multiple testing to the resulting set of p‐values (BH correction: Benjamini & Hochberg, 1995).

Next, we also explored the general condition of backcross versus intra‐subspecific pairings, by pooling data for the two backcross pairs (i.e., RO and OR pairs) and ran two Welsh *t* tests on log‐transformed total sperm data to test for differences between backcross and RR pairs only and pooled data for both intra‐subspecific pair types (i.e., RR and YY pairs). Figures were constructed using *yarrr* (Phillips, 2017), and modeling assumptions (normality and heterogeneity of variance of residuals) were assessed visually (following Zuur, Ieno, Walker, Saveliev, & Smith, 2009). All tests were two‐tailed and considered significant at α < 0.05. Data presented are mean ± *SD *unless otherwise noted.

## 3 RESULTS

**Figure 1:**
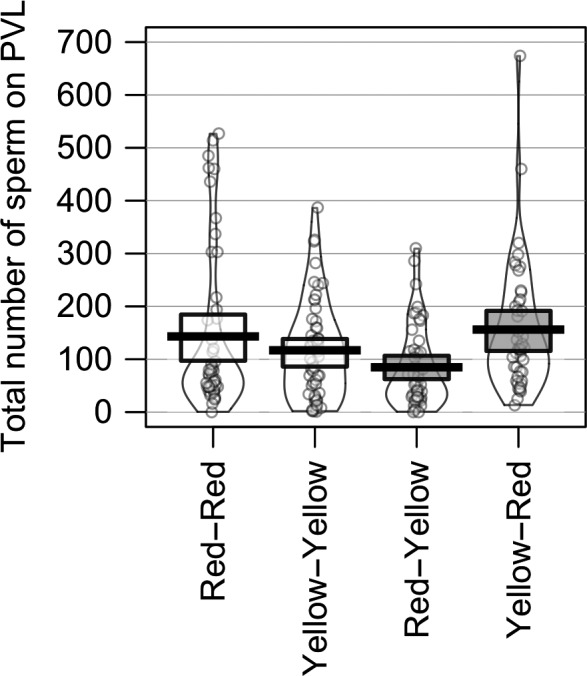
Total number of Sperm on the PVL in intra‐subspecific (white fill: red or yellow only pairs) and inter‐subspecific pairs (light gray fill: one red and one yellow individual). Pairs did not significantly differ, but before BH correction inter‐subspecific pairs significantly differed. Female color is listed first. Circles represent raw data points with bean smooth density outline, bar denotes average, box shows 95% high‐density interval

In our comparison of PVL sperm numbers in intra‐ and inter‐subspecific pair types (Table 1, Figure 1), we found no difference in total PVL sperm per egg between the two intra‐subspecific pair types (RR vs. YY; Table 2). Similarly, neither inter‐subspecific cross (i.e., RY, YR) differed from the intra‐subspecific YY pairs nor the intra‐subspecific RR pairs (Table 2). Finally, RY pairs did not differ from YR pairs in terms of total sperm reaching the egg; though for this last comparison, we note that prior to BH correction, YR pairs had a significantly higher number of PVL sperm relative to RY pairs (Table 2). The main effect of clutch was not significant (Table 2).

**Table 1:** Total number of sperm on the PVL by color pairing. Female color is listed first. Pure pooled combines YY and RR pairs, backcross pooled combines RO and OR pairs

	Total sperm			*n*	
Color pairing (Female First)	Mean ± *SE*	Min per egg	Max per egg	Egg	Clutch
Red‐Red	143.2 ± 21.8	0	527	49	10
Yellow‐Yellow	116.9 ± 12.8	1	387	53	12
Red‐Yellow	84.8 ± 11.0	0	310	48	12
Yellow‐Red	156.1 ± 18.3	13	674	45	10
Red‐Orange	79.5 ± 16.3	1	660	63	12
Orange‐Red	71.9 ± 5.8	3	239	56	10
Intra‐Pooled	129.6 ± 12.4	0	527	102	22
Backcross pooled	75.9 ± 9.0	1	660	119	22

**Table 2:** The GLMMs for impact on average PVL sperm per egg for different color pairings of red (R) and yellow (Y) or red and orange (O) long‐tailed finch individuals. These GLMMs used random factors of female and male identity and the random effect of pair identity with random intercepts as well as slopes by using an interaction with the order effect of clutch. As reference level color was changed for post hoc comparisons of all pair types, intercepts are given for each. Original p‐values were BH corrected *p*‐values to correct to multiple testing false discovery rate

Reference level color	Fixed effect	Estimate	Standard error	*z*‐value	Original *p*‐value	BH *p‐*value
Red and yellow model						
RR	(intercept)	4.5	0.37	12.01	0.001	–
RR	YY	0.04	0.5	0.08	0.94	0.94
RR	YR	0.52	0.38	1.35	0.18	0.49
RR	RY	−0.51	0.51	−1.02	0.31	0.51
YY	(intercept)	4.54	0.34	13.31	0.001	–
YY	YR	0.48	0.5	0.97	0.33	0.51
YY	RY	−0.55	0.38	−1.45	0.15	0.50
YR	(intercept)	5.02	0.38	13.38	0.001	–
YR	RY	−1.04	0.51	3.01	0.04	0.22
	Clutch	−0.02	0.17	−0.14	0.89	0.94

	Variance	*SD*	*n*	Corr		ICC
Marginal R2	0.10					
Pair ID	0.40	0.63	22			0.28
Clutch	0.49	0.70		−0.42		
Male	0.31	0.56	12			0.20
Female	0.42	0.24	12			0.69
				Conditional *R* ^2^		0.54

Reference level color	Fixed effect	Estimate	Standard error	*z*‐value	Original *p*‐value	BH* p*‐value
Red and orange model						
RR	(intercept)	4.44	0.24	18.17	0.001	–
RR	OR	−0.32	0.35	−0.9	0.37	0.51
RR	RO	−0.69	0.14	−4.8	0.001	0.01
OR	(intercept)	4.12	0.28	14.71	0.001	–
OR	RO	10.37	0.34	−1.08	0.28	0.51
	Clutch	0.08	0.18	0.42	0.672	0.82

	Variance	*SD*	*n*	Corr		ICC
Marginal R2	0.07					
Pair ID	0.09	0.30	16	−1.00		0.00
Clutch	0.40	0.64				
Male	0.33	0.57	10			0.00
Female	0.00	0.00	13			0.40
				Conditional *R* ^2^		0.44

**Figure 2:**
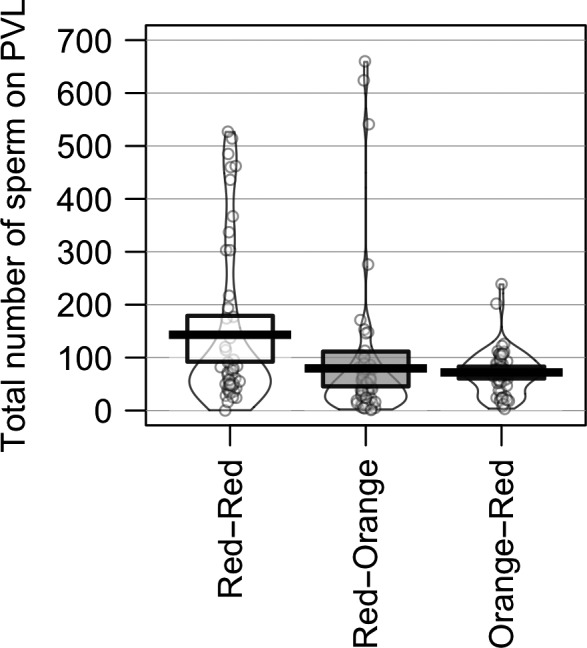
Total number of Sperm on the PVL in backcrossed versus intra‐subspecific parental matings (female color first): intra‐subspecific red pairs (white fill) significantly differed from backcrossed (mid‐gray fill) red‐orange and orange‐red pairs when pooled, and from red‐orange on their own. Circles represent raw data points with bean smooth density outline, bar denotes average, box shows 95% high‐density interval

In contrast, we found that backcross pairs had lower PVL sperm numbers relative to the intra‐subspecific pair (Table 1; Figure 2). Specifically, RO pairs had significantly lower PVL sperm numbers compared to the intra‐subspecific RR pair type (Table 2). Furthermore, while pairwise comparisons showed no difference between OR and RR pairs (Table 2), there was also no difference between the backcross pair types (RO vs. OR; Table 2). Therefore, to explore the more general instance of backcross matings, we pooled data for the two backcross pair types and compared PVL sperm numbers between backcross pairs and intra‐subspecific pairs. Overall, backcross pairs had significantly fewer sperm reaching the egg than RR pairs (*t* = 3.03, *p* = 0.003), and pooled intra‐subspecific pairs (*t* = 3.11, *p* = 0.009). In the model, the main effect of clutch was not significant (Table 2).

## 4 DISCUSSION

Sexual reproduction is a complicated process; between copulation and zygote formation, numerous morphological, physiological, and molecular interactions between male and female proteins, cells, and tissues are critical to successful fertilization. Our results demonstrate that the number of sperm that reach the ova differ according to experimental pair type in the long‐tailed finch. Specifically, we found that when pooled, backcross matings result in a 41.4% and 47% (intra‐subspecific pooled and RR pairs only, respectively) reduction in average numbers of sperm reaching the PVL of the ova compared to the intra‐subspecific mating (Table 1). We consider our findings in light of the putative fertility costs of low PVL sperm numbers and the potential such postcopulatory processes may have for gene flow and reproductive isolation in these subspecies.

In birds, successful fertilization requires multiple sperms to penetrate the inner PVL of the ovum and a low number of sperm on the PVL is associated with low fertilization success (Birkhead & Fletcher, 1998; Mizushima, 2017). Thus, our result indicates that F1 backcross mating pairs likely experience reduced fitness relative to intra‐subspecific pairs. In future work, we would recommend that before sampling eggs to remove the PVL, they should be incubated for about a day to assess successful fertilization (following Hemmings & Birkhead, 2015). Such an approach would have allowed us to determine the likely reduction in fertilization success that would be driven by a >40% reduction in PVL sperm. However, in the absence of such data, it seems plausible that it could affect fertilization. This is because the number of sperm on the PVL differs significantly across a number of similarly sized passerines, suggesting that there is an optimal number for each species (Hurley et al., 2017). Thus the >40% reduction in the number of PVL sperm found in the backcrossed pairings will presumably be suboptimal, and as such may constitute a PMPZ barrier reducing the mating success of such pairings relative to pure pairs.

Such a barrier would constrain gene flow between the two subspecies and is consistent with the relatively narrow hybridization zone that exists in the wild where the two subspecies are currently in secondary contact (Griffith & Hooper, 2017). Similar selection against F1 hybrid individuals backcrossed with parental species has been shown to drive reproductive isolation in a diversity of systems (e.g., Harper & Hart, 2005, Lijtmaer, Mahler, & Tubaro, 2003). Recent work across a range of taxa suggests that a major contributing factor to reduced reproductive capacity of F1 hybrid/parental crosses is the misregulation of nuclear and mitochondrial gene expression (e.g., eels (Jacobsen et al., 2017); rabbits (Rafati et al., 2018); sparrows (Runemark et al., 2018; Trier, Hermansen, Sætre, & Bailey, 2014), leading to some fertility issues (e.g., copepods (Ellison & Burton, 2008); sparrows (Eroukhmanoff et al., 2016; Trier et al., 2014).

Given the complex and cryptic nature of ejaculate‐female/sperm‐egg interactions in internally fertilizing species, understanding the mechanism underlying differential success in sperm reaching the PVL among the different pair types is difficult. In birds, there are several proposed stages that could serve as potential PMPZ barriers to hetero‐specific sperm (reviewed in Birkhead & Brillard, 2007), including failure of sperm to: (a) traverse the vagina, (b) enter or exit female sperm storage tubules (SSTs), (c) be transported from SSTs to the infundibulum, the site of fertilization, (d) penetrate the PVL, and (e) fuse with the female pronucleus. However, we lack the necessary information to determine which of these potential barriers may be acting in our backcross mating conditions. For the inter‐subspecific pairs, however, one explanation worth considering is that the small differences in PVL sperm numbers between YR and RY pairs might be linked to differential sperm storage. Specifically, given that the length of sperm and female SSTs correlate with birds (Briskie & Montgomerie, 1993) and that the subspecies exhibit significant differences in sperm lengths (Y males have longer sperm than R males; Rowe, Griffith, et al., 2015), it is possible that the lower number of PVL sperm in RY pairs could be explained by poor storage (e.g., of long sperm in short SSTs, compared to YR pairs with short sperm stored in long SSTs). We acknowledge, however, that the differences in sperm length between the subspecies is small and, given that SST length is likely to be somewhat variable within and among females (Briskie & Montgomerie, 1993), it is unclear if such differences are sufficient to cause the patterns observed in our study. Nonetheless, differential sperm storage linked to variation in sperm size has been observed in experimental studies of the zebra finch (Hemmings & Birkhead, 2017), suggesting that consideration of the role of sperm morphology in sperm‐female/sperm‐egg interactions is warranted.

Variation in number of copulations, not controlled for in this study, could also account for a reduction of sperm in backcrossed pairs (Birkhead, Hunter, & Pellatt, 1989). However, females appear to have a great deal of control over the number of sperm reaching the egg, perhaps especially when sperm numbers are low (Hemmings & Birkhead, 2015). Further, it seems unlikely that the observed reduction in PVL sperm numbers in the backcross pairs is simply the result of reduced fertility of hybrid (O) males. This is because, while RO pairs resulted in a significant decline in PVL sperm numbers (44.5% decrease), OR pairs also exhibited a reduction in similar magnitude (49.8% decrease; Figure 2) in the average total PVL sperm per egg, even though it wasn't significant.

In addition to the examination of the role of sperm variation, another goal worthy of future research effort would be an exploration of the potential role of reproductive proteins (e.g., proteins in the sperm and egg, as well as the seminal fluid) in mediating ejaculate‐female/sperm‐egg interactions. Reproductive proteins are generally thought to play a significant role in reproductive barriers (Swanson & Vacquier, 2002), but our knowledge of reproductive proteins is taxonomically limited. In domesticated fowl, seminal fluid proteins (Borziak, Alvarez‐Fernandez, Karr, Pizzari, & Dorus, 2016) and extracellular ions (e.g., calcium and sodium: Froman & Feltmann, 2005) have been shown to influence sperm swimming speed. Moreover, in passerine birds, both sperm and seminal fluid of males are likely to contain numerous proteins linked to sperm viability and function, as well as sperm‐egg interactions. As such, it is reasonable to assume that molecular mechanisms may play a role in determining the patterns of differential sperm usage observed here.

While somewhat preliminary due to the scale of our experiments, our findings provide empirical evidence for a mechanism that may contribute to a PMPZ barrier operating in an avian species and provide the impetus for further work to examine potential behavioral, morphological, physiological, and molecular mechanisms determining the extent to which sperm make their way to the site of fertilization in different pairing contexts.

## CONFLICT OF INTEREST

None declared.

## AUTHOR CONTRIBUTION

LLH, SCG, and MR conceived and designed the experiment. LLH, MR, and SCG wrote the manuscript. LLH conducted the empirical work. LLH and MR conducted statistical analysis.

## DATA ACCESSIBILITY

Dataset is available at the Knowledge Network for Biocomplexity (https://doi.org/10.5063/F1N29V51).
